# Translating Clinical Research to Clinical Care in Nephrology: A Qualitative Study of Nephrology Clinicians

**DOI:** 10.1016/j.xkme.2022.100459

**Published:** 2022-04-01

**Authors:** Dana Ravyn, Beth Goodwin, Rob Lowney, Arlene Chapman

**Affiliations:** 1 CMEology, West Hartford, CT; 2 Section of Nephrology, Department of Medicine, Institute for Translational Medicine, University of Chicago, Chicago, IL

**Keywords:** Autosomal dominant polycystic kidney disease, continuing education, health care environment, learning theory, nephrology, qualitative analysis, research translation

## Abstract

**Rationale & Objective:**

The translation of clinical research to practice has been the subject of intense scrutiny in the efforts to identify ways to improve the uptake of findings that can enhance patient care.

**Study Design:**

This study evaluated the experience of nephrology health care providers who manage patients with autosomal dominant polycystic kidney disease (ADPKD) to identify promoters and barriers to the translation of research results into clinical practice. We used inductive thematic analysis to evaluate the experience, attitudes, and beliefs of physicians in the evaluation and translation of research findings into clinical practice for the care of patients with ADPKD.

**Setting & Participants:**

Participants in a continuing education activity on ADPKD volunteered for semistructured interviews exploring their experience translating new knowledge into care for patients with ADPKD. An independent institutional review board (Solutions IRB) found the study to be exempt as an educational survey.

**Analytical Approach:**

Transcripts were coded and excerpted, and emergent themes and relationships were identified through an analysis performed using Dedoose software. Particular attention was paid to characterizing the facilitators and barriers to research translation at different levels of the health care environment.

**Results:**

Textual interpretation of data from 13 interviews showed that while well-established barriers to research translation are prevalent among health care providers managing patients with ADPKD, these clinicians also face unique challenges. Principal among these is the burden of interpreting the clinical research literature given the lack of official guidelines.

**Limitations:**

This study did not explore the translation of all levels of research, such as basic science and animal studies, and it was limited to the translation of knowledge from clinical studies. The number of participants was limited but was found to be sufficient for saturation.

**Conclusions:**

We identified factors that may either enhance or impede research translation for nephrology health care providers. These observations may help in the design of continuing education interventions to promote innovation.

**Plain-Language Summary**

Despite progress in clinical research, translating new findings about autosomal dominant polycystic kidney disease in patient care can be slow. We conducted semistructured interviews to evaluate the experiences of nephrologists in the evaluation and translation of research findings. Inductive thematic analysis was used to qualitatively analyze the data. The results showed that barriers to research translation are prevalent among nephrology clinicians and that those managing patients with autosomal dominant polycystic kidney disease face unique challenges, such as a lack of official guidelines. Factors that can either enhance or impede research translation were identified. These observations may help in the development of continuing education interventions to promote innovation.

Research has shown that translational science exists on a continuum beginning with biomedical science, progressing to studies in animals and humans, and eventually being applied to clinical practice.^1^^,^^2^ Various models have characterized the process of translation as being nonlinear, such as having a circular or a back-and-forth process of change while ultimately progressing between research and practice. Translating knowledge to practice is challenging because of the complexity of health care systems and processes underlying the adoption of new practices in medicine.^3^ Accordingly, change in clinical practice is often frustratingly slow, and it can take as long as 17 years for new findings to be put into use.^4^

Understanding translation is important to identify the skills needed to use research results to improve patient care. Additionally, identifying and mitigating barriers to research translation can enhance the efficiency of bringing new knowledge into practice and enhancing the diffusion of innovation.^5^ Barriers and facilitators of research translation are heterogeneous with regard to disease states, and they are influenced by a myriad of organizational, social, and structural factors.^6^ Although linear and nonlinear models have been used as a framework to examine research implementation, little is known about how health care providers evaluate and implement research findings in practice. Qualitative studies that take a holistic and nonlinear approach to this question by considering the magnitude and influence of contextual factors are needed.

Autosomal dominant polycystic kidney disease (ADPKD) is the fourth most common cause of kidney failure in the United States,^7^, ^8^, ^9^ where it is estimated to affect 140,000 people^9^ and leads to significant morbidity, with total costs of $7.3 to $9.6 billion annually.^10^ ADPKD is considered an orphan disease, and the numbers of patients available to participate in clinical studies are limited. Outside of academic or research settings, the infrequency of ADPKD in nephrology practice limits clinicians’ experience with and exposure to the most current evidence-based practices.^11^ Translational research in ADPKD has expanded rapidly in recent decades. Clinicians apply recent clinical findings in molecular genetics, imaging, and disease-modifying therapy to better manage patients with ADPKD by diagnosing the disease earlier, promptly initiating and tailoring treatment, and potentially improving outcomes.^12^

To gain insight into the facilitators and barriers to translation of research into practice as specifically related to ADPKD, we conducted a qualitative study of health care providers practicing nephrology. Nephrology clinicians were interviewed to better understand their experiences evaluating and applying new findings to the care of patients with ADPKD.

## Methods

The online continuing medical education (CME) learning activity, *Strategies to Improve Management of ADPKD: Navigating Pitfalls and Overcoming Challenges*, was released in September 2020 and was available online for 12 months at Healio.com and freeCME.com. The activity was certified for physicians and nurses by Medical Education Resources, a nonprofit accredited provider, and the faculty person was one of the authors (AC). The target audience was physicians and nurses involved in the care of patients with ADPKD. On completion of the activity evaluation, participants were asked if they wanted to volunteer to participate in an interview on the translation of clinical results to patient care.

The qualitative study described here was designed to enhance the understanding of research translation in the nephrology setting by identifying emerging themes. We employed an inductive strategy to analyze the results of semistructured interviews. Participants were identified when they volunteered for an interview at the time of completing the CME activity. Informed consent was obtained verbally, and a semistructured interview was conducted by telephone by one investigator (DR). Interview questions embraced several methods, including hypothetical (Do you think that…?), provocative (It’s believed that…what do you think?), ideal (What would be a solution to…?), and interpretive (What do you mean by…?). The interview was dynamic and focused on the experiences, opinions, feelings, and knowledge of the interviewee while soliciting input from the participant. Survey questions were partially refined based on the interview results, but the conceptual framework of the interviews was preserved throughout data collection. Interviews were conducted up to the point of redundancy of responses and a lack of emergent themes.

Audio recordings were transcribed verbatim. Deidentified transcripts were read, and a framework was constructed considering both explanatory and exploratory observations. Codes were formulated by 2 investigators (DR and BG); codes were then reviewed and revised by all investigators. To a limited extent, sensitizing concepts from extant research were used to create the initial coding schemes used to validate the conceptual framework of the study.^13^ Excerpts were identified and assigned to established codes. Interviews and notes were coded and analyzed using Dedoose software version 9.0.^14^ Thematic analysis was initially performed by DR and BG, then reviewed and revised by all the investigators.

The nature and risks/benefits of participation were explained to all prospective interviewees, and all participants provided verbal consent before the interviews. All participant data were deidentified. The study was reviewed by an independent institutional review board (Solutions IRB), and the research was verified to be an exempt educational survey according to 45 CFR 46.101(b)(2).

## Results

A total of 1,926 health care professionals participated in the CME learning activity, including 462 nephrologists. Half of the participants had been practicing for more than 20 years. The self-reported confidence levels of the learners increased substantially following the exercise, and a statistically significant improvement was seen in knowledge when the pretest and posttest questions were evaluated (n = 3; *P* < 0.001 for all). Thirteen participants volunteered for an interview. Interviewees were comparable to the population of CME activity participants in terms of practice setting, experience, age, and gender. All the 13 participants who completed the semistructured interview were nephrology providers. Twelve interviewees were nephrologists and 1 was a nephrology nurse practitioner.

Textual analysis of the interview data revealed several factors related to ADPKD research evaluation and translation that emerged as individual or shared ideas. Individual responses were heterogeneous and suggested a wide range in the level of comfort and success in evaluating and applying clinical research data to the management of ADPKD.

### Challenges

Several recurrent themes were noted that are common to translational science in many areas of medical practice. These include the quality and quantity of research, institutional and noninstitutional structural factors, dissemination of findings, practice type, and sociocultural characteristics of the work environment.^6^^,^^15^ The challenges identified in the present study included poor comprehension of complex research reports, difficulty with statistics, and the lack of an organizational culture that promotes the uptake of new research results. As expected, these factors diverged substantially when comparing different practice settings. In academic settings, nephrologists were often in the vanguard of research translation, whereas translation lagged in private and community practices where the demands of patient care responsibilities often impeded the evaluation and implementation of new approaches to ADPKD care.

One clinician explained, “…[T]hat’s not what the private practice is about at all…you go in, you see a lot of patients, you try to follow the standards of care…I’m trying to stay up-to-date with the most recent treatment options…but there is a big difference between [me and] an academic person practicing…you don’t just go and read literature.” (MD) Another stated, “You know, when you’re in that academic environment, people constantly push you to look at the data, and I’m not sure that happens as much in private practice. So if there could be simpler algorithms for people to follow, I think that would be helpful.” (MD)

### Burden of the Literature

With the absence of official guidelines and consensus recommendations for ADPKD, the private practitioner faces the burden of trying to determine best-care practices from original articles and reviews, which are technically challenging and time-consuming to understand. As one interviewee shared, “When you’re in private practice, you don’t have time or expertise to understand all the details of an original manuscript…and that makes it really granular for them to know how to deal with this.” (MD)

A key observation in the thematic framework analysis of this study was that several factors can either impede or promote research translation (Table 1). This “boon or bust” pattern clearly emerged as a recurrent theme reflecting shared experiences among respondents.

**Table 1:** Factors That Can Promote or Impede Research Translation into Clinical Practice in ADPKD

Factor	Impedes Translation	Promotes Translation
Research methods	• Statistics can be hard to interpret or may be done improperly • Research translation is often not considered when designing studies	• A statistical result such as a *P* value can reassure the clinician • Author conclusions about clinical implications help readers
CME	• Hard to find time • Content may not be in the area of interest	• Often relevant and offers an opportunity to learn from experts • Can validate current practices
Dissemination	• Clinicians may not recognize authors/coauthors and may not be able to conclude anything about their reputation for research • Nonacademic institutions may not have journal clubs or grand rounds • Many clinicians do not have time or resources to devote to conferences and meetings • Some journals lack a highly favorable reputation	• Recognizing someone with a good reputation on an article adds to the sense of validity • Data from a highly rated journal can reassure the clinician
Institution	• Many institutions impede innovation because of limited resources or commitment • Competing priorities	• Institutions can help evidence-based practice thrive by promoting interdisciplinary or communication strategies
Years in practice	• Senior clinicians may be relying on outdated approaches • Less senior clinicians may want to do everything by the book and not know how to be flexible in their care practices	• Less senior clinicians are often more up-to-date and interested in newer ways of doing things
Type of practice	• Nonacademic disadvantages, including lack of collegiality, journal clubs, grand rounds, etc • Competing priorities • Clinicians in nonacademic settings may be behind on the newest therapies and approaches	• Being in an academic setting is generally seen as more conducive to research translation than private practice
Guidelines	• No official promulgated guidelines for ADPKD • Many are too long and complex; may contradict one another	• Clinicians in nonacademic settings rely heavily on guidelines to determine the current standard of care
Innovation	• Sometimes clinicians resist new ideas and methods	• Newer ways of practice are based on fresh data and the most recent studies
Time	• Clinicians know that new findings are important but may only skim or not stay abreast of the literature • Some clinicians cannot afford a subscription to proprietary sources of up-to-date practice information	• Clinicians use online and mobile sources of concise and practical content, relying on author credibility • Mobile digital strategies can help clinicians search and learn more efficiently

Abbreviations: ADPKD, autosomal dominant polycystic kidney disease; CME, continuing medical education.

### Interpretation of Statistical Data

One of the most prominent factors in research translation that emerged from the interview data is difficulty in comprehending research methods. Statistics can be challenging to interpret, and many participants found them to be vexing when trying to assimilate study results. One participant noted, “I always found myself very challenged by biostatistics, but along the way, I’ve been taught enough to at least have a primitive sense of are the right questions being asked, is the data being looked at in a reasonable way…” (MD) Another stated, “I think that the hardest part to understand of a study is probably the statistics and all the math involved; that sometimes loses one, especially. So I would say that’s what distracts the most…” (MD) Another clinician observed, “People have concerns that the statistics may not have been done properly in the first place, but they don’t know how to figure that out…” (MD)

Some statistical results can clarify the interpretation of the clinical implications and provide confidence in the adoption of new practices. For example, results such as a significant *P* value, the number needed to treat, and the number needed to harm offer practical guidance for clinicians.^16^

There was a recurring sentiment that clinicians are sometimes unable to critically evaluate research articles and identify the implications for care from individual studies. As one interviewee noted, “Some of our colleagues probably haven’t been trained in a setting where they’ve literally been taught to think critically to evaluate papers.” (MD) Another participant reflected, “…people are not interpreting these [studies] because maybe they understand the data, but they just don't know really how to apply it and put it together; what does it really mean for patient care?” (MD) Another participant suggested, “I think there should be a bit more education on research. Polycystic [kidney disease] is not so well talked about. We talk about diabetes and hypertension and cancer and stroke, but not much about polycystic kidney disease.” (NP)

### Clinical Translation Is Often Not Considered When Reporting Results

Another recognized barrier to translation into clinical practice is that research translation is not considered when designing or interpreting clinical research results, making it hard for the reader to discern the clinical implications of the study. Authors of clinical research often take a conservative approach when making conclusions about the implications of their results. As one participant observed, “Sometimes the researchers don’t want to step out on a limb, and even though they think they know what the promise is of their research, they're afraid to say it ‘out loud’…I think what they could do is speculate on what the outcomes might be, and indicate what their reservations might be, and that lets people look at things in a new way.” (MD)

There are relatively few studies in the field of ADPKD, and the lack of replication made some clinicians reluctant to adopt new practices. Replicated study findings carry greater weight as evidence supporting practice changes.

### Reputation of Journals and Authors

The importance of the dissemination of research in innovation was another key finding of this study. The provenance of research was highly influential for learners. Data from a highly rated journal reassured clinicians, whereas they were more skeptical about the implementation of studies published in less prestigious journals. Similarly, the reputation of the study authors added to the sense of validity on the part of health care providers. One participant described their thought process as, “What journal am I seeing it in? Is it a top-notch general journal…is it one of the best of the renal journals?” (MD) Another participant would ask, “Is it people I know and respect over a period of time where there’s a certain trust in integrity, and that I know they’ve run medium to large outcomes trials before?” (MD)

Many nephrology health care providers in private and community practice did not have the time or resources to attend conferences and meetings. CME was commonly cited as a dominant factor in understanding the implications of research for clinical practice. Study participants repeatedly emphasized the unique role that CME played in their approach to innovation. One clinician noted, “CME programs help…they’re opening your mind up to other treatments, other possibilities, increasing your knowledge rather than this little narrow path that you follow.” (MD) Another clinician observed, “In general, [providers] found [CME] activities they had completed to be highly relevant and a valuable opportunity to learn from experts for both adopting new practices and validating current practice.” (MD) Another participant reflected, “I think the information [from this activity] was really objective, and I think it helps to clarify the current studies that are out there. So it kind of summarizes everything.” (MD)

### Practice and Institution Characteristics

Institutional and practice characteristics weighed heavily on whether research translation was impeded or promoted. The promotion of communication and interdisciplinary strategies to improve care can encourage innovation.^17^ Many participants felt that their institutions impeded innovation because they had competing priorities or lacked resources for implementation, or their administration was not committed to the adoption of new approaches.

Differences in seniority and experience among nephrologists were also found to be decisive factors in research translation. Participants reported that senior providers may continue to rely on outdated approaches. In contrast, less senior clinicians are often more up-to-date on ADPKD research and are interested in newer ways of doing things but can be reluctant to rely on their experience and instincts because they often seek the security of strictly adhering to guidelines. As one participant described, “Sometimes it’s hard to change [younger physicians’] minds. They want to do what is in the book. It’s hard to make them sort of be open to other ideas. But someone who has been practicing for 20 years…they’re more open to other things.” (MD)

## Discussion

This qualitative study evaluated data from nephrology health care providers who manage patients with ADPKD to characterize their experience evaluating and applying clinical research to patient care. There are well-known gaps in the application of research findings to clinical practice, although little is known about these gaps as they relate to ADPKD care practices by nephrology providers.

A landmark US study found that adults received recommended care only 55% of the time^18^ and that the delivery of appropriate care decreased in the presence of comorbid conditions.^19^ More recently, the CareTrack study evaluated the appropriateness of the health care delivery provided to 1,154 adults and found that compliance with indicators of appropriate care varied from 13%-90% depending on the medical condition.^20^ Given the lack of widely accepted clinical guidelines and quality indicators for the management of ADPKD, it is difficult to assess the appropriateness and currency of evidence-based care being delivered. Clinicians treating patients with ADPKD must rely heavily on expert opinion and consensus for treatment decision-making. As a result, it was not surprising to find a wide range of attitudes, beliefs, and practices in ADPKD research translation among nephrology clinicians.

A key finding of this study was the “boon or bust” theme, in which the same factors could act as either facilitators or barriers to research translation in different situations. It is possible that this phenomenon reflects the lack of official evidence-based promulgated guidelines for the management of ADPKD. We postulate that in the absence of clear indicators of appropriate care, the nephrology health care provider has the burden of navigating the research to determine best practices in caring for patients with ADPKD. In lieu of official practice guidelines, evaluating factors such as the methods, statistical analysis, quality, implications, and dissemination of research becomes the burden of the nonacademic practicing clinician. In this scenario, the success of implementation can easily pivot on factors such as author and journal reputation, terminology, comprehension of statistical methods, the learner’s seniority, the limitations of the research design, and knowledge of the ADPKD canon. Further, guidelines are designed to save time and effort in the implementation of best practices. In the absence of such resources, health care providers must draw on scarce resources, such as time to review research, attendance at conferences or journal clubs, and guidance from colleagues. In response to these demands, health care providers frequently depend on CME to learn about recent developments in ADPKD and how to apply findings to clinical care. Other strategies identified as important in this process included digital and mobile resources and concise online practice summaries written by highly credible authors.

Continuing education is well suited for disseminating new practices and promoting practice innovation.^21^, ^22^, ^23^ Methods such as transformative learning, team-based learning, and simulations can facilitate the diffusion of innovation and clinical knowledge development.^24^, ^25^, ^26^ Interview participants cited the importance of learning about research implications and innovations in the CME activity that they completed as part of this study.

There was a wide spectrum of attitudes toward preparedness for research translation on the part of nephrology providers. The transtheoretical model of change proposes 6 stages of change that characterize an individual’s readiness to adopt behavior change (precontemplation, contemplation, preparation, action, maintenance, and termination).^22^ Like Rogers’s diffusion of innovation theory,^27^ the transtheoretical model postulates several stages of diffusion in the application of new knowledge and understanding. We found in a previous study that CME participants recognize the mechanisms of behavior change that are important for translational mechanisms in evidence-based care for patients with multiple sclerosis.^28^

**Figure 1:**
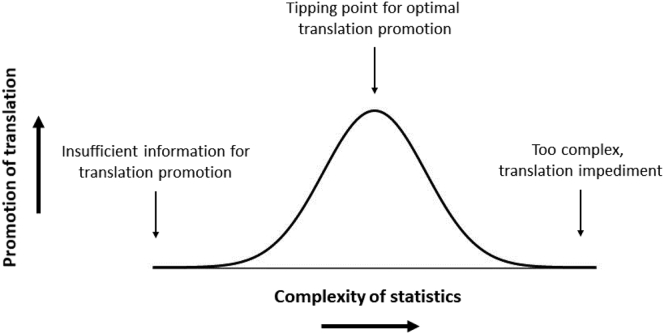
Model of a postulated tipping point characterizing the effectiveness of various factors in the promotion of research translation. In this example, the learner seeks statistical data to interpret implications for care. Understanding the implications for care is difficult if the statistical data are inadequate (left). Optimally, some statistical findings such as *P* values, number needed to treat, and number needed to harm can promote understanding and make translation more likely (center). When statistics pass the tipping point beyond which they become overly complex and difficult to understand clinically, the factor becomes an impediment to the translation (right).

Identification of the factors that can be either a promoter or an impediment to the translation of ADPKD research can inform strategies to improve the diffusion of innovative practices. Consideration of these factors may be beneficial when formulating education and other interventions to encourage the translation of clinical research into the care of patients with ADPKD. Notably, consideration of pivotal factors reveals tipping points, beyond which factors become more (or less) likely to be effective at promoting the translation of research findings. For example, statistical data can help clinicians interpret the clinical implications of research results, but they must be presented in such a way so as not to pass the tipping point beyond which they become overly complex and impede the learner’s understanding (Fig 1).

The results reported here should be viewed in the context of the study limitations. Our research did not consider issues related to all stages of translation, such as the translation of basic science, biomedical advances, and animal studies. This study focused on what has been referred to as T2 translation, or “the translation of results from clinical studies into everyday clinical practice and health decision-making.”^1^

Although a relatively small number of health care providers were interviewed, redundancy in data collection was observed. A sufficient level of saturation was likely achieved with the 13 interviewees. Our sample was highly homogeneous, and we only sought the power to detect prevalent themes, factors that improve the likelihood of identifying themes in a smaller sample.

One study assessed the necessary sample size for thematic analysis of interviews conducted with patients with multiple sclerosis.^28^ The study authors concluded that 12 interviews were adequate for saturation. In another study, the researchers conducted 60 interviews and observed saturation after the first 12 interviews.^29^ In the present study, a narrative method was used, which emphasizes illustrative or evocative sampling, rather than data saturation.^30^

Although many factors influencing research translation identified in this study have been characterized in the literature on translational science, the context was intended to focus on the nephrology setting, and in particular, on the care of patients with ADPKD. Despite this observation, the results of the analysis were not intended to be generalizable or universal but rather reflective of the participants who practice in the area of focus. Future research is needed to extend these results to other areas of clinical practice, such as other disease states in nephrology or other orphan diseases. This and subsequent research in this area may provide insights into the influence of CME and other educational and noneducational interventions regarding translation practices and patient outcomes in ADPKD.

In conclusion, various factors that were influential in the translation of clinical research into patient care among nephrology providers managing patients with ADPKD were identified. Overall, this thematic analysis helps identify potential educational and noneducational interventions to enhance promoters and mitigate barriers to research translation. This study found that the same factors can be either promoters or impediments to translation and that understanding the specific considerations that influence this difference in effect—herein referred to as the tipping point—may help in formulating strategies to improve translation in the nephrology setting.
